# Hospital and Institutional News

**Published:** 1914-09-05

**Authors:** 


### HOSPITAL AND INSTITUTIONAL NEWS.

### OUR EDUCATIONAL NUMBER.

Our readers will realise that the altogether exceptional pressure on our space caused by the publication of our Special Educational Number at a time when war news is being sent to us from so many hospitals and centres of work has been very difficult to cope with. Thus the Institutional Worker Section has had unavoidably to be held over, and in addition much war news, which our special correspondents have kindly sent, has also been crowded out. We hope that they will realise the difficulty, however, and show that they have done so by making an extra effort on receiving this issue to ensure that next week The Hospital shall contain a complete summary of all the latest news. In the present Special Number we have thought it well once more to devote special attention to the medical careers afforded by the Army and Navy Medical Services, while a contributor of wide experience affords a fascinating glimpse into the opportunities of a medical man in India for what he calls "a life worth living." The question of private practice is one which at the present critical period is exercising the minds both of intending practitioners and of those already in practice, and is well worth study in the light of such available facts as may make reasonable deductions possible.

### THE FIRST WOUNDED TO ARRIVE.

During last week-end the first batches of wounded began to arrive in this country from Belgium and France. Apart from those who have been received at the Royal Herbert Hospital, Woolwich, and at Plymouth and Bishop's Stortford (for Cambridge), others have been received at Brighton, Portsmouth, and Birmingham. Apparently, so far the numbers sent to each place have varied between 120 and 300, which last number are also reported as having been admitted to the London Hospital. Thanks to Mr. Alfred Salmon, a member of the committee, a number of vans were in readiness at Waterloo to meet the trains from Southampton, which has been the centre to which, in the first instance, the wounded are transferred. Members of the London staff met the trains and superintended the removal of the wounded, and since the most serious cases are dealt with at the Royal Victoria Hospital, Netley, speaking generally, no injuries likely to prove fatal or permanently to incapacitate the sufferers were among the arrivals. It is interesting to note that so many of the wounds are in the legs, owing to the bursting of shells behind the men who had taken cover. In this respect the direction of the German fire by aeroplanes is worth notice. The hospital rule against smoking has been relaxed at the London in favour of the wounded, who are, in the most part, in high spirits, notwithstanding their injuries. In the provincial centres alluded to motor-cars were much in evidence at the stations, and everywhere alike the welcome given to the wounded was warm in the extreme.

### A HOSPITAL AS A RECRUITING STATION.

On the evening of August 28 a public meeting was held opposite the entrance of the North Riding Infirmary, Middlesbrough, for the purpose of encouraging enlistment in Lord Kitchener's new army. At the conclusion of a stirring address by Councillor Spence, the young men in the crowded audience were invited to come forward as recruits. Although a number of persons had been engaged to take the names and particulars, it was found that the number offering themselves was greater than could be dealt with in the public streets. The intending recruits were therefore asked to pass through the infirmary gates and line up in the carriage-way. Mr. Charles Postgate, the secretary-superintendent of the infirmary, who was on the spot, came to the rescue of the promoters of the meeting by having the commodious out-patient waiting-hall immediately prepared, where the whole of the recruits were received and dealt with. The enrolment of the recruits presented a busy scene, in which a number of gentlemen were engaged for more than an hour, the secretary-superintendent taking a hand in the work himself. Within the space of one hour over 150 recruits were enrolled, and as they passed by the wards of the infirmary the patients who were able to get to the windows gave them rousing cheers.

### VACCINATION AND THE ARMY.

It has often been remarked that disease is more deadly to armies than rifle and cannon fire, and it is interesting to note already the suggestions which have been made in regard to preventive methods. In our own columns the question of anti-typhoid inoculation has recently been raised, and since supported publicly by Sir William Osler, who urges compulsory vaccination, and points out that "pending the issue of a compulsory order, it is the duty of the medical officers of the Territorial Force to urge" the importance of the measure on as many men as possible. Last week we drew attention to the proposed corps for cholera service with the Russian forces, and in the House of Commons Mr. Tennant, Under-Secretary for War, has stated in reply to questions that, while the commanding officers had been circularised to the effect that the men under their command with conscientious objections should not be vaccinated, every effort was made to persuade the men to undergo it on the ground that unless they submitted they were not likely to be of much service in the field. Certainly this consideration places "conscientious objectors" in a very difficult situation, and it will be interesting to see what effect it has upon their numbers and determination.

### A SOUTH AFRICAN SECRETARY ON HOSPITAL CONSTRUCTION.

English hospital officers will turn with interest to a pamphlet entitled "Hints on Hospital Construction and Equipment," by Mr. Cyril Benson, the resident secretary at Pretoria Hospital, South Africa. The pamphlet, only thirty pages long, is intended to cover, "as far as possible, all the ground necessary for consideration by a body responsible for the erection and equipping of a hospital," and arose out of a draft report presented by the writer for the building sub-committee which was appointed to consider the plans for the new Pretoria Hospital. Necessarily simple in aim it contains principles like the following: "Each building erected on an unsatisfactory site makes the claim for a new hospital on another site more difficult to be recognised"; where climate and covered approaches allow "it is better to accommodate nurses in a separate building." The writer argues that the German system of construction must involve many administrative difficulties on account of size, distance, and decentralisation of departments. His own institution, he says, is planned somewhat similarly to University College Hospital, London. The ground-floor plan he describes as follows: From the administrative block two corridors run—one S.E. and the other S.W.—to circulating halls, from which the wards extend V shape N.E. and S.E. on one side and N.W. and S.W., with a cut-off passage between each ward block and the circulating hall. Another corridor runs E. and W. some distance behind the administrative block, and this also joins the circulating halls by extensions N.E. and N.W. In this corridor the special departments are accommodated on one side and the out-patient department on the other, all being close to the service and patients' lifts.

### A HANDBOOK FOR COMMITTEE-MEN.

Moreover, such points in ward construction as windows and walls are then briefly touched on, and a few interesting notes are devoted to plans of native hospitals. Ward equipment, from ward waggons to bedside calls are then briefly described, and the pamphlet concludes with a few points on other details of equipment and furniture. Though the hospital officer of experience will hardly gain much from the pamphlet, it should be invaluable for hospital committee-men, and especially for new members, who, while having no time to spare to study the long and mostly foreign text-books, would be interested in and profit by a brief introductory pamphlet of this kind. Copies can, no doubt, be obtained from the Pretoria Hospital.

### THE ASYLUMS BOARD AND THE NAVY.

Not everyone realises the enormous scope of the activities of the Metropolitan Asylums Board. The "institution" of which the public is least aware, but which does probably the most consistent and permanent good to its "inmates," is the training-ship *Exmouth*. This vessel takes care of about 700 boys, of whom about 300 per annum are entered into the Navy or mercantile marine. The annual reports of the Captain-Superintendent show the excellence of the training and the satisfactory start in life which are given to the boys. Last year was marked by the launching of a sea-going training-ship under the name of the *Exmouth II.* We imagine that there can be no two opinions as to the value of the practical rounding-off of the training on the parent ship which the sea-going vessel now enables the Board to give to the lads entrusted to its care. Curious lack of support is nevertheless received from Boards of Guardians in London. Only about half the entries during the year were drawn from Metropolitan parishes; on the other hand, country Boards continue to evince a desire to enter into agreements for the training on the *Exmouth* of boys chargeable to them, and never fail to take advantage of their agreements in sending their best boys to the ship. And moreover, in supplying every year well-trained boys to the Royal Navy and mercantile marine, the ship performs a valuable service to the State.

### THE OUTLOOK FOR HOSPITAL INCOME.

While various forecasts on this subject reach us there is no doubt that when the accounts of the voluntary hospitals for the present year are made up the deviation of charity from the hospitals to the National War Fund will account for a certain loss. A correspondent, for example, points to the fact that during the last few years many thousands of pounds have been added to hospital funds as the result of practice matches, and many hospitals have practically regarded the donations as a permanent part of their income. Another adverse effect of the war will probably be caused by the consequent unemployment and the feared increase in cost of food, from which the Hospital Saturday and Sunday Funds and the other organisations, through which the working classes contribute to the hospitals, will suffer. An instance of this is to hand from Walsall, where, in accordance with his usual custom, Mr. Pat Collins gave one day's takings at his Fête at Bloxwich for the benefit of the Walsall and District Hospital. Although the weather was glorious and the attractions well up to the average, the proceeds fell short of the previous year's by one quarter.

### THE WAR AND MEDICAL QUALIFICATION.

To meet the situation arising out of the war the Royal Colleges of Physicians and Surgeons are announced to have decided that a special final examination for the conjoint diploma shall begin on September 8. All candidates who are eligible for admission to the October examination will be considered eligible for admission to it, and those who were referred for three months at the July examination will also be admissible to the special examination in September without producing certificates of further study. It is also understood that candidates who fail at the special examination starting next week may be admitted to re-examination in October by resolution of the examiners on consideration of the standard attained. This special war examination for medical men, as it may be termed, is of interest in view of the recent proposals to "speed up" the process of qualification, though it is far less drastic and indeed revolutionary than were the suggestions which gained currency a few weeks ago.

### WAR ACCOMMODATION IN SALFORD.

The War Office have accepted, if required, the offer of the board of management of the Salford Royal Hospital to place sixty beds in the first instance, and ultimately one hundred, at the disposal of the wounded. With this end in view, the honorary medical and surgical staff having agreed to treat such patients, the additional beds necessary are to be obtained, and the nursing staff is to be increased. It is understood that the hospital's usual work will continue, and that provision has been made for the treatment of urgent cases and accidents. Thus the Manchester district, in view of previous offers, appears to be well provided for, and though now that there has been a month of hostilities we are in a better position to gauge the numbers of wounded likely to be received, and to predict that the struggle will be as protracted and the casualties quite as heavy as were feared, there should be no doubt that sufficient accommodation is available. The problem to be solved is rather in the allocation of the wounded to different districts and institutions, once the naval and military hospitals are fully occupied, and that is a matter which circumstances will chiefly influence.

### ARRIVAL OF WOUNDED AT CHATHAM.

Our special correspondent for the Chatham district reports that fifteen of the crew who were injured in the H.M.S. *Amphion* disaster were removed to the Royal Naval Hospital, Chatham. They are reported to be progressing favourably. In addition a number of German wounded prisoners are lying at Fort Pitt Hospital, Chatham. The men, many of whom are suffering from terrible injuries, were rescued from the mine-layer *Koenigin Luise*.

### EMERGENCY CALL AT GRAVESEND.

The Gravesend Hospital has received notice that it has been affiliated to the Central Military Hospital at Chatham, and will form a section of that hospital. Last Saturday morning news was received by telephone to have sixty beds ready for immediate use. These were got ready very quickly, but up to the time of writing no patients have arrived. The arrangements which the local Voluntary Aid Detachment are making proceed satisfactorily.

### SCHOOL MEDICAL OFFICERS ON ACTIVE SERVICE.

In consequence of the Board of Education understanding that in a number of areas school medical officers and others engaged in the school medical service will be giving their services to the Navy or Army, and that in consequence difficulties in carrying on the work may be experienced, a circular to local education authorities has been issued. This says in effect that the school medical service must be maintained with the least possible interruption, and that the Board relies upon the local education authorities to make good working arrangements for carrying on the work of those medical officers absent through the war. While the Board of Education does not offer any general suggestions as to what temporary steps should be taken, it states that every allowance will be made for the difficulties, and, provided that the existing work be carried on with reasonable efficiency, it will accept for the purposes of their own administration the best arrangements which can be made during the emergency. This making of local authorities responsible, and undertaking to judge them mainly by results, is enforced by a request for the names of those temporarily absent, and of the arrangements made to supply their place.

### LOCAL GOVERNMENT BOARD ADVICE ON DRUG CONTRACTS.

A number of Poor-Law authorities have received advice from the Local Government Board with reference to their drug contracts and the effect of the war upon them, referred to in the last issue of The Hospital (page 592). Writing to the Gloucester Board of Guardians, they state that the Board should arrange with the contractors to supply the goods contracted for as far as possible on terms to be settled later on, when both parties have further information to guide them. But in a communication to the Berwick Board of Guardians the authorities at Whitehall say that they are disposed to the view that the war does not affect the liability of contracts, but in cases where contractors suggested that they could not continue to supply goods at contract prices, local authorities should continue the contracts on the understanding that while the contract prices would form the basis of arrangements, the actual amounts would be settled later on by agreement or by arbitration. In the columns of The Hospital on the 29th ult. we suggested the adoption of a similar course to that referred to in the latter part of the letter of the Local Government Board to the Berwick Board of Guardians.

### INFIRMARIES AND SCHEMES OF WORK.

In view of our Note last week on infirmary superintendents and schemes of public work, it is instructive to learn that the Wandsworth Board of Guardians are pushing forward an extension of their St. James's Hill Infirmary, on which it is proposed to expend a sum of £10,160, with a view to finding work for those thrown out of employment through the war. The difficulty about this and any other building scheme taken in hand by Boards of Guardians at present is the lack of building materials in London. The strike in the building trade has been over for some time now, and there is an abundance of work in the Metropolis for the men; but the bulk remain idle as the railway companies since the war began have refused to carry building materials. The Camberwell Board of Guardians are considering the advisability of putting in hand the painting work required at their infirmary, which in the ordinary course of events would not be done for another twelve months. Other Boards are expediting similar work in order to find employment. Could not action be taken by the Government to ensure that building material be removed by the railway companies when their lines are not required for the removal of troops and war stores?

### THE BOLTON GUARDIANS' NEW BUILDINGS.

Mr. James Bowling, the vice-chairman of the Bolton Board of Guardians, performed last week the opening ceremony of the important extensions which have been undertaken to the Townleys Hospitals. The male hospital consists of a block, two storeys in height, which provides accommodation for 130 beds. Half a block each is also devoted to male and female phthisical patients, and the construction of these is chiefly distinguished by such additional facilities for open-air treatment as enlarged balconies and verandahs. These two buildings, which are also two storeys high, provide accommodation for sixty-four beds apiece. Built of local brick, with facings of Yorkshire stone and of terra-cotta, the extensions correspond with the adjoining institutional buildings. Reference may also be made to the Hollins Cottage Homes, which consist of detached cottages and a school built in 1879. Of this group, apart from additions, including a superintendent's house, the school itself has been converted to hospital purposes. Thus an important series of additions, each designed, it would seem, to secure complete classification, has been carried out, and the opening of the extension of the Townleys Hospitals marks an important stage in local institutional life and work.

### A GOLD MEDAL FOR ARMY SANITATION.

One or two prizes by laymen have already been offered for various individual feats of arms, but one much more remarkable on the part of institutional workers is made by the Chadwick Trustees for a feat in connection with Army sanitation and hygiene. The proposal of the Chadwick Trustees is embodied in the following resolution: "That in view of the immense importance of encouraging in every way the promotion of careful sanitary organisation in the Naval and Military Services during the present campaign, the Chadwick Trustees have resolved, under the powers conferred upon them under the scheme they administer, to announce their intention to award at the close of this year the Chadwick Gold Medal and £50 each to the naval and military medical officer respectively in the British Service who shall have distinguished himself most in promoting the health of the men in the Navy and the Army. The nomination for such presentations to be, as provided by the terms of the trust, by the Directors-General of the Naval and Military Medical Services respectively." It will be observed that no specific action is here insisted on, but the award seems intended to fall to that naval or military officer who has shown himself most alert in looking after the health of his men. Thus initiative and thoroughness combined are here singled out for honour, and it must be admitted that no service is more signal than that which prevents the spread of disease in camp. The award, it will be noticed, is to be made "at the close of this year," by which time, at any rate, the effects of any oversight or omission will have made themselves felt, and the absence of illness would vouch for the high standard of the Army sanitation in the field.

### THE MANUFACTURE OF FOREIGN DRUGS.

There has been little change in the position since last week, although the advancing tendency has become more pronounced in the case of certain drugs. The action of the authorities in obtaining information as to stocks of articles of commerce and in taking possession of any such articles unreasonably withheld should effectively prevent the cornering of drugs. Good is also to be expected as the result of the appointment of a committee (consisting of the president, vice-president, and secretary of the Pharmaceutical Society, and Mr. C. A. Hill and Mr. John C. Umney) to furnish information to the Government in regard to the current prices of drugs, with particular reference to cases in which prices can be shown to be artificially inflated as the result of speculative transactions or the unreasonable withholding of stocks from the market. The question of manufacturing some of the drugs which have hitherto been obtainable solely from Germany is also being seriously considered by our manufacturers, and, although there are technical difficulties in the way, there is reason to hope that these will be overcome in some cases at least. The desire to extend our industry in fine chemicals and synthetic drugs is, of course, being encouraged by the Government, one of the measures taken in this direction being the enactment of new patent legislation which empowers the Patent Office to grant licences to British subjects to use patents of which the licensees are Germans or Austrians. But the development of this industry must of necessity be slow at first.

### THE NAVY AND THE DRUG MARKET.

Besides the drugs mentioned last week many others have advanced in price to a serious extent. A few articles quotations for which have been raised are the following:—Sugar of milk, santonin, belladonna and gentian roots, atropine, caffeine, camphor, cream of tartar, Epsom salts, chamomiles, senna, lithia salts. But these are only a small proportion; indeed, most drugs are dearer. Any further advance in the price of bromides is not expected at the moment, but present quotations are at least double the normal. Cantharides and ergot of rye are much dearer, as Russia is the principal source of supply. Mercurials are only slightly dearer. Cod-liver oil has not advanced to any serious extent, and this fact is a splendid testimonial to the vigilance of the British Navy in the North Sea.

